# Nicotinamide N‐methyltransferase inhibition improves limb function in experimental peripheral artery disease

**DOI:** 10.14814/phy2.70615

**Published:** 2025-10-18

**Authors:** Gengfu Dong, Jaewon Choi, Yufen Li, Diana C. Muller, Zhuoxin Li, Yangyi E. Luo, Terence E. Ryan

**Affiliations:** ^1^ Department of Applied Physiology and Kinesiology The University of Florida Gainesville Florida USA; ^2^ Center for Exercise Science The University of Florida Gainesville Florida USA; ^3^ Myology Institute The University of Florida Gainesville Florida USA

**Keywords:** ischemia, metabolism, skeletal muscle, vascular disease

## Abstract

Peripheral artery disease (PAD) impairs limb perfusion, walking ability, and increases the risk of amputation. Although current therapies reduce cardiovascular events, few interventions improve skeletal muscle function in PAD. Nicotinamide adenine dinucleotide (NAD^+^) metabolism is disrupted in PAD. Thus, it was hypothesized that inhibition of nicotinamide N‐methyltransferase (NNMT), an enzyme that diverts precursors from the NAD^+^ salvage pathway, would improve ischemic limb function. We analyzed NAD^+^ pathway expression in gastrocnemius muscle from patients with and without PAD using RNA sequencing and proteomics datasets. Single‐cell RNA sequencing data were used to assess NNMT expression in muscle stem cells (MuSCs) from BALB/cJ and C57BL/6J mice following hindlimb ischemia (HLI). Male BALB/cJ mice (*n* = 24) were randomized to either placebo or a NNMT inhibitor (NNMTi) delivered 3 h prior to HLI and daily thereafter. Functional assessments included laser Doppler perfusion imaging, muscle contractility, and a 6‐min limb function test. Histological analyses were used to assess myofiber area and capillary density. NNMT mRNA and protein levels were significantly elevated in skeletal muscle from patients with PAD and were persistently elevated in MuSCs from BALB/cJ mice after HLI. NNMTi treatment did not affect limb perfusion recovery or capillary density but trended toward reduced necrosis severity (*p* = 0.08). Muscle mass and myofiber size were unchanged by treatment; however, NNMTi significantly improved muscle strength (*p* < 0.0001), power (*p* = 0.0305), and total work (*p* = 0.0367) in ischemic limbs compared to placebo. Inhibition of NNMT enhanced ischemic muscle strength and performance in a preclinical model of PAD independent of changes in perfusion.

## 1 INTRODUCTION

Atherosclerotic cardiovascular disease (ASCVD) remains a major cause of mortality. Peripheral artery disease (PAD) is one form of ASCVD in which reduced blood flow to the lower limbs causes impaired walking ability and increases the risk of amputation and mortality (Aday & Matsushita, 2021; Hammond et al., 2021). Tremendous progress has been made to reduce mortality from cardiovascular events stemming from ASCVD via the usage of antiplatelet, antithrombotic, and cholesterol‐lowering medications. However, treatments to improve lower limb function or walking performance in PAD remain limited. Beyond cilostazol, a phosphodiesterase inhibitor that induces vasodilation, only supervised exercise therapy has been shown to improve walking performance in patients with PAD (Treat‐Jacobson et al., 2019). Accumulating evidence has documented a significant skeletal muscle pathology in patients with PAD (Anderson et al., 2009; Cluff et al., 2013; England et al., 1992; Ferrucci et al., 2023; Ismaeel et al., 2022; McDermott et al., 2007; McDermott, Ferrucci, et al., 2009; McGuigan et al., 2001; Mietus et al., 2020; Pass et al., 2023; Pipinos et al., 2003; Ryan et al., 2018), yet there are still no treatments to improve muscle health and performance in PAD.

Impaired skeletal muscle metabolism has been well documented in the lower limb of patients with PAD (Anderson et al., 2009; Hands et al., 1986; Hart, Layec, Trinity, Kwon, et al., 2018; Hart, Layec, Trinity, Le Fur, et al., 2018; Hiatt et al., 1996; Ismaeel et al., 2022; Kim et al., 2025; Park et al., 2022; Pipinos et al., 2000; Pipinos et al., 2003; Ryan et al., 2018; Schocke et al., 2008; Wilburn et al., 2024). These observations span a wide range of experimental methodologies including in vivo phosphorus magnetic resonance spectroscopy to ex vivo measures in muscle biopsy specimens where oxygen levels are not limiting. The agreement between these methodologies underscores the high fidelity of these findings and places a spotlight on muscle mitochondria in PAD. However, specific biochemical mechanisms underlying impairments in skeletal muscle mitochondrial function are not fully understood. A recent study identified the microRNA miR‐210, which is increased by hypoxia, as a negative regulator of muscle mitochondrial function in patients with PAD (Ismaeel et al., 2022).

Nicotinamide adenine dinucleotide (NAD^+^) is a vital coenzyme involved in redox reactions, mitochondrial function, and signaling pathways that regulate muscle health and regeneration. In skeletal muscle, NAD^+^ levels decline with age, injury, and metabolic stress, impairing the function of sirtuins and other NAD^+^‐dependent enzymes that are critical for mitochondrial biogenesis, stem cell maintenance, and repair (Frederick et al., 2016; Goody & Henry, 2018; Zhang et al., 2016). Restoration of NAD^+^ levels through precursors such as nicotinamide riboside (NR) or nicotinamide mononucleotide (NMN) has been shown to enhance mitochondrial function and improve regenerative capacity in preclinical models (Ryu et al., 2016; Zhang et al., 2016), but some studies have questioned whether the same effects are present in humans (Dollerup et al., 2020). In fact, a recent study in mice reported lifelong depletion of NAD does not alter skeletal muscle aging (Chubanava et al., 2025). However, a recent double‐blind, randomized controlled trial reported that NR supplementation improves walking performance in patients with PAD (McDermott et al., 2024). Emerging evidence has established that NAD metabolism is highlight sensitive to oxygen levels (Burtscher et al., 2025). In the context of PAD—where aging, disuse, and ischemic injury converge—enhancing NAD^+^ availability may support muscle regeneration through improved mitochondrial function and metabolic resilience. Several published studies provide support for this notion. For example, Frederick et al. (Frederick et al., 2016) demonstrated that NAD^+^ depletion impairs muscle regeneration, and supplementation with NAD^+^ precursors rescue satellite cell function and myofiber repair. Similarly, Zhang et al. (2016) reported that NR administration boosts NAD^+^ levels in aged mice, improving muscle stem cell activity and muscle regeneration. Moreover, NAD^+^ metabolism is tightly linked to the activity of SIRT1 which regulates the epigenome of muscle stem cells through its actions as a histone deacetylase, thereby regulating regenerative capacity (Ryall et al., 2015). These findings underscore the importance of maintaining NAD^+^ homeostasis for muscle regeneration, and they highlight therapeutic opportunities to target NAD^+^ pathways in ischemic and degenerative muscle diseases.

In this study, we identified significantly higher expression of nicotinamide N‐methyltransferase (NNMT), an enzyme involved in the methylation of nicotinamide, thereby diverting it from the NAD^+^ salvage pathway (Dimet‐Wiley et al., 2024; Liang et al., 2024; Neelakantan et al., 2019), in skeletal muscle of patients with PAD. Thus, we sought to test whether treatment with an inhibitor of NNMT could improve limb function in mice with experimental PAD.

## 2 METHODS

### 2.1 Human muscle analyses

We analyzed the expression of genes involved in NAD^+^ homeostasis in the gastrocnemius muscle of patients without PAD (non‐PAD) and severe PAD (chronic limb threatening ischemic, CLTI) using a previously published RNA sequencing dataset (Ryan et al., 2018). Protein expression of NNMT was quantified from a proteomic analysis performed in a separate cohort of patients without PAD (non‐PAD) and severe PAD (chronic limb threatening ischemic, CLTI) (Ryan et al., 2022). Patient characteristics and clinical information are available in the original publications (Ryan et al., 2018; Ryan et al., 2022).

### 2.2 Single cell RNA sequencing analysis

We obtained a publicly available single‐cell RNA sequencing (scRNAseq) dataset published by Southerland et al. (2023). The processed data was loaded in Scanpy (https://scanpy.readthedocs.io/en/stable/index.html) in Python, and the expression of *Nnmt* in muscle stem cells (MuSCs) was compared between stains using a Welch test.

### 2.3 Animals

Experiments were conducted on male 12‐week‐old BALB/cJ (Stock No. 000651, *n* = 19) mice purchased from Jackson Laboratories. All mice were housed in temperature (22°C) and light‐controlled (12:12‐h light–dark) rooms and maintained on standard chow (Envigo Teklad Global 18% Protein Rodent Diet 2918 irradiated pellet) with free access to food and water prior to enrollment. All animal experiments adhered to the *Guide for the Care and Use of Laboratory Animals* from the Institute for Laboratory Animal Research, National Research Council, Washington, D.C., National Academy Press. All procedures were approved by the Institutional Animal Care and Use Committee of the University of Florida (protocol 202400000402). Both the researchers and surgeon were blinded to the genotype and/or group of the animals.

### 2.4 Animal model of peripheral artery disease

Femoral artery ligation (Berru et al., 2019; Ryan et al., 2016) was performed by anesthetizing mice with intraperitoneal injection of ketamine (100 mg/kg) and xylazine (10 mg/kg) and surgically inducing unilateral hindlimb ischemia (HLI) by placing silk ligatures on the femoral artery just distal to the inguinal ligament and immediately proximal to the saphenous and popliteal branches. Extended‐release buprenorphine (3.25 mg/kg, EthiqaXR, Fidelis Animal Health) was given preoperatively for analgesia. Mice that experienced foot necrosis continued to receive extended‐release buprenorphine until euthanasia. The extent of limb necrosis was recorded as follows: grade 0, no necrosis in ischemic limb; grade I, necrosis limited to the toes; grade II, necrosis extending to the dorsum pedis; grade III, necrosis extending to the crus; grade IV, necrosis extending to mid‐tibia or complete limb necrosis.

### 2.5 Placebo and NNMTi treatments

We obtained NNMTi, a potent inhibitor nicotinamide N‐methyltransferase (NNMT), from MedChemExpress (Cat No. HY‐131042). NNMTi was dissolved in dimethyl sulfoxide (DMSO), diluted with sterile saline, and to mice via intraperitoneal injections. A placebo control involving equal volume of DMSO diluted with saline was used. NNMTi was delivered to mice at 10 mg/kg beginning 3 h prior to HLI surgery and once daily thereafter. Both the surgeon and experimenters were blinded to the treatment conditions until the completion of all measurements and analysis.

### 2.6 Limb perfusion assessment

Limb perfusion recovery was measured using a high‐resolution Laser Doppler Imager (moorLDI‐HDR, Moor Instruments) while mice were anesthetized with ketamine/xylazine. The hindlimb of the mice was imaged in the prone position at a 512 × 512‐pixel resolution, and regions of interest were drawn around the entire paw to quantify perfusion. Perfusion values were expressed as a percentage of the non‐surgical control limb for all images.

### 2.7 Nerve mediated isometric muscle contraction

Functional tests of the plantar flexor muscles, specifically the plantarflexor complex (gastrocnemius/soleus/plantaris), were measured in situ using a whole animal system (model 1300A, Aurora Scientific Inc., Aurora, ON, Canada). Mice were anesthetized with an intraperitoneal injection of ketamine (100 mg/kg) and xylazine (10 mg/kg). The plantarflexor complex from the ischemic limb was isolated from its distal insertion, leaving the vasculature intact. The distal portion of the *Achilles* tendon was tied using a 4‐0 silk suture attached to the lever arm of the force transducer (Cambridge Technology; Model No. 2250). Muscle contractions were elicited by stimulating the sciatic nerve via bipolar electrodes using square wave pulses (Aurora Scientific, Model 701A stimulator). The Lab‐View‐based DMC program (version 615A.v6.0, Aurora Scientific Inc.) was used for data collection and servomotor control. After obtaining optimal length via twitch contractions, an abbreviated force‐frequency curve was performed. Isometric contractions were elicited using a 500 ms train (current 2 mA, pulse width 0.2 ms) at stimulation frequencies of 1, 40, 80, and 150 Hz, with 1 min of rest provided between contractions. Tetanic force levels were reported as absolute force and specific force (absolute force normalized to muscle mass). The peak tetanic force generated from the 80 Hz contraction was used as the reference force for subsequent isotonic contractions.

### 2.8 6‐min limb function test

To evaluate muscular performance across a series of repetitive contractions, we utilized afterloaded isotonic contractions which involved stimulating the nerve with supramaximal voltage (2 mA, 0.2 ms pulse width, 100 ms train duration) at 80 Hz and allowing the muscle to shorten once it exceeded 30% of the 80 Hz peak isometric force. This facilitates quantification several characteristics including force (newtons), displacement (mm), shortening velocity (m/s), mechanical power (watts), and mechanical work (joules). Shortening velocity was quantified as the change in distance from a 10 ms period which began 20 ms after the initial length change. Mechanical peak power was calculated as the product of the shortening velocity (m/s) and corresponding force. Instantaneous velocity as the first derivative of the position‐time tracing $\left(v\left(t\right)=\frac{\mathrm{dy}}{\mathrm{dt}};\text{where}\;y\;\text{is position and}\;t\;\text{is time}\right)$ which was then used to calculate instantaneous power as the product of the instantaneous velocity and the corresponding force. Finally, we quantified mechanical work for each contraction as the integral of the instantaneous power‐time tracing (Work = ∫Instantaneous Power·dt; where t is time). To sum of mechanical work performed in each contraction was reported as the total work, a measure akin to the distance covered in a 6‐min walk test which is most used in clinical trials with PAD patients. During the 6‐min test, laser Doppler flowmetry was employed by placing the probe on the medial gastrocnemius throughout the 6‐min test to assess functional hemodynamics continuously through the test. All testing was performed on a temperature‐controlled platform to maintain a body temperature of 37°C. This test was thoroughly validated in several experimental models in a recently published study (Palzkill et al., 2025).

### 2.9 Immunofluorescence microscopy

Following completion of the 6‐min limb function test, the plantarflexor complex (gastrocnemius, soleus, plantaris muscles) was carefully dissected, weighed, embedded in optimal cutting temperature (OCT) compound, and frozen in liquid nitrogen‐cooled isopentane. Animals were then euthanized by cervical dislocation under anesthesia. Using a Leica 3050S cryotome, 10 μm‐thick transverse sections of the gastrocnemius muscle were cut and mounted on microscope slides. All muscle sections were fixed with 4% paraformaldehyde (ThermoFisher Scientific, Cat. No. J19943‐K2) for 10 min, then three washes with 1× phosphate buffered saline (PBS), and slides were incubated in blocking solution (5% goat serum + 1% BSA in 1xPBS) for 2 h. Muscle sections were incubated overnight at 4°C with a primary antibody against laminin (Millipore‐Sigma, Cat. No. L9393, 1:100 dilution) to label the basal lamina surrounding myofibers. The following morning, sections were washed three times with 1xPBS and stained with Alexa Fluor 488 goat anti‐rabbit IgG (ThermoFisher Scientific, Cat. No. A‐21121, 1:250 dilution) secondary antibody and 1 mg/mL Griffonia simplicifolia lectin (GSL) isolectin B4, Dylight 594 (Vector Laboratories; Cat. No. DL‐1207) to fluorescently label α‐galactose residues on the surface of endothelial cells of capillaries for 1 h at room temperature, followed by three more washes with 1xPBS. Coverslips were mounted onto all slides using Vectashield hardmount (Vector Laboratories, Cat. No. H‐1500). Slides were imaged at 20× magnification with an Evos FL2 Auto microscope (ThermoFisher Scientific), and tiled images of the entire section were obtained for analysis. Images were thresholded and the number of capillaries was quantified by a blinded investigator using a particle counter in Fiji/ImageJ. Skeletal myofiber cross‐sectional area (CSA) was quantified using MuscleJ2 (Danckaert et al., 2023), an automated analysis software developed in ImageJ/Fiji.

To quantify embryonic myosin heavy chain fibers, muscle sections were incubated overnight at 4°C with a primary antibody against laminin (Millipore‐sigma, cat. No. L9393, 1:100 dilution) and embryonic myosin heavy chain (Myh3, developmental studies hybridoma Bank, University of Iowa, cat. No. F1.652, 1:20 dilution). The following morning sections were washed three times with 1xPBS and stained with Alexa Fluor 488 goat anti‐rabbit IgG (ThermoFisher scientific, cat. No. A‐21121, 1:250 dilution) and Alexa Fluor 647 goat anti‐mouse IgG1 (ThermoFisher scientific, cat. No. A‐21240, 1:250 dilution) secondary antibodies. Coverslips were mounted onto all slides using Vectashield hardmount (vector laboratories, cat. No. H‐1500). Slides were imaged at 20× magnification with an Evos FL2 auto microscope (ThermoFisher scientific) and tiled images of the entire section were obtained for analysis. Images were analyzed by a blinded investigator by counting embryonic myosin positive fibers and normalizing the count to the total muscle area in Fiji/ImageJ.

### 2.10 RNA isolation and quantitative polymerase chain reaction

All muscles were lysed with TRIzol reagent (Invitrogen, Cat. No. 15‐596‐018) using a PowerLyzer 24 bead mill homogenizer (Qiagen). Total RNA was purified with the Direct‐zol RNA MiniPrep Kit (Zymo Research, Cat. No. R2052) according to the manufacturer's instructions. LunaScript RT SuperMix Kit (New England Biolabs, Cat. No. E3010L) was used for cDNA generation. Quantitative PCR was performed using the Luna Universal qPCR Master Mix (New England Biolabs, Cat. No. M3003X) with SYBR Green‐based detection on a QuantStudio 3 Real‐Time PCR System (ThermoFisher Scientific). Relative gene expression was calculated using the 2^−ΔΔCT^ method with expression normalized to the respective control group of each experiment. The following primers were used: *L32*: Forward‐TTCCTGGTCCACAATGTCAA, Reverse‐GGCTTTTCGGTTCTTAGAGGA; *Ppargc1a*: Forward‐TATGGAGTGACATAGAGTGTGCT, Reverse‐CCACTTCAATCCACCCAGAAAG; *Myh8*: Forward‐GGAGAGGATTGAGGCCCAAAA, Reverse‐CACGGTCACTTTCCCTCCATC; *Nnmt1*: Forward‐ATCTTGAAGGCAACAGAATGAAGG, Reverse – TCCTGAGGGCAGTGCGATAG; *Sirt1*: Forward‐GGCCTAATAGACTTGCAAAGGA, Reverse‐CTCAGCACCGTGGAATATGTAA; *Sirt3*: Forward‐TACAGAAATCAGTGCCCCGA, Reverse‐GGTGGACACAAGAACTGCTG.

### 2.11 Immunoblotting

Snap frozen muscle tissue was homogenized in RIPA lysis buffer (ThermoFisher Scientific, Cat. No. 89900) supplemented with 1:100 Protease Inhibitor (Millipore Sigma, Cat. No. P8340) and 1:100 Phosphatase Inhibitor (Millipore Sigma, Cat. No. 324627) in glass Teflon homogenizers and centrifuged at 10,000*g* for 10 min at 4°C. The supernatant was collected, and protein quantification was performed using a bicinchoninic acid protein assay (ThermoFisher Scientific; Cat. No. SL256970). 2× Laemmli buffer (BioRad, Cat. No. 161‐0737) and β‐mercaptoethanol (ACROS; Cat. No. 60‐24‐2) were added to the samples, which were incubated in boiling water for 5 min. 10 μL of a pre‐stained ladder (BioRad, Cat. No. 1610394) was loaded in the first lane of a 4%–20% Criterion TGX Stain‐Free Protein Gel (BioRad, Cat. No. 4568095), along with 40 μg of muscle lysate from each sample. Gel electrophoresis was run at 100V and then imaged for total protein on a BioRad imager (BioRad Chemidoc) before transferring to a polyvinylidene fluoride (PVDF) membrane using a BioRad Trans Blot Turbo system. The PVDF membrane was then imaged for total protein and incubated in blocking buffer (Licor, Cat. No. 927‐60001) for 1 h at room temperature while rocking. The membrane was incubated overnight at 4°C with total OXPHOS rodent western antibody (1:1000 dilution, Abcam ab110413) in blocking buffer to determine relative levels of OXPHOS complex subunits in mouse. After overnight incubation, the membranes were washed 3 × 5 min with TBS + 0.01% tween‐80. The membranes were then incubated for 1 h in blocking solution with secondary antibody (Licor, Cat. No. 926‐68070, 1:8000 dilution) to detect OXPHOS proteins. Next, the membranes were then washed 3 × 5 min in TBS + 0.01% tween and imaged on a BioRad Chemidoc imager. All blots and images shown in the results are uncropped and uncut. Quantification of band intensity normalized to the total protein was performed using BioRad's Image Lab software.

### 2.12 Statistical analysis

All data are presented as the mean ± standard deviation (SD). Normality of data was tested with the Shapiro–Wilk test and inspection of QQ plots. Data involving comparisons of two groups were analyzed using a Student's *t*‐test when normally distributed and a Mann–Whitney test when normality could not be assessed. When making comparisons with repeated measures, data were analyzed using repeated measures analysis of variance (ANOVA) with Tukey's post‐hoc test or two‐way ANOVA when appropriate. Comparison of the proportion of mice with foot necrosis scores was made using a chi‐squared test. All statistical testing was conducted using GraphPad Prism software (version 9.0, https://www.graphpad.com) or Python. Bulk RNAseq and proteomic data were compared using a Mann–Whitney test. scRNAseq data were compared using a Welch test. In all cases, *p* < 0.05 was considered statistically significant.

## 3 RESULTS

### 3.1 NAD ^+^ salvage pathway is mis‐regulated in muscle from patients with PAD

**FIGURE 1:**
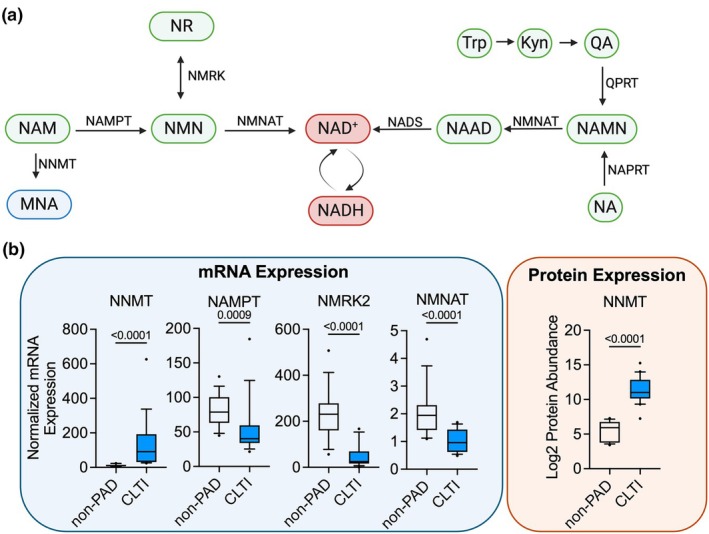
NAD^+^ salvage pathway is misregulated in muscle from patients with PAD. (a) Graphical depiction of the NAD^+^ biosynthesis and salvage pathway made with Biorender.com. (b) Quantification of mRNA expression (*n* = 16 non‐PAD and *n* = 15 CLTI) of genes involved in NAD^+^ salvage. Quantification of protein expression (*n* = 10 non‐PAD and *n* = 10 CLTI) of NNMT. Data analyzed using the Mann–Whitney *U* test and presented as boxplots with 90% confidence intervals. Kyn, kynurenine; MNA, methylnicotinamide; NA, nicotinic acid; NAAD, deamido‐nicotinamide adenine dinucleotide; NAD^+^, oxidized nicotinamide adenine dinucleotide; NADH, reduced nicotinamide adenine dinucleotide; NADS, nicotinamide adenine dinucleotide synthase; NAM, nicotinamide; NAMN, nicotinate mononucleotide; NAMPT, nicotinamide phosphoribosyltransferase; NAPRT, nicotinic acid phosphoribosyltransferase; NMN, nicotinamide mononucleotide; NMNAT, nicotinamide mononucleotide adenylyltransferase; NMRK, nicotinamide riboside kinase; NNMT, nicotinamide N‐methyltransferase; NR, nicotinamide riboside; QA, quinolinic acid; QPRT, quinolinate phosphoribosyltransferase; Trp, tryptophan.

Considering that a recent randomized clinical trial has reported promising results for improving walking function in patients with PAD following 6 months of NR treatment (Ferreira et al., 2023), we first wanted to explore if pathways involved in regulating NAD^+^ homeostasis (Figure 1a) are misregulated in the muscle of patients with PAD. We first examined RNA sequencing data collected from non‐PAD (*n* = 15) and severe PAD patients with chronic limb‐threatening ischemia (CLTI, *n* = 16) which has been previously published (Ryan et al., 2018). Patients with CLTI displayed significantly higher mRNA expression of *NNMT*, whereas *NAMPT*, *NMRK*, and *NMNAT* were significantly downregulated in CLTI compared with non‐PAD (Figure 1b). These data agree with data published by Ferrucci et al. (Ferrucci et al., 2023) who reported downregulation of genes involved in NAD^+^ homeostasis in the gastrocnemius muscle of patients with PAD. Next, we quantified NNMT protein expression from a published proteomics dataset on non‐PAD participants and patients with PAD (Ryan et al., 2022). This analysis confirmed that the protein abundance of NNMT was also significantly higher in patients with CLTI compared with non‐PAD controls (Figure 1b). It is worth noting that patients with PAD often present with risk factors or comorbid conditions such as diabetes, smoking, kidney disease, and others, which likely influence NAD metabolism independent of the limb blood flow.

### 3.2 NNMT expression is persistently elevated in muscle stem cells from the ischemia sensitive BALB/cJ mice

**FIGURE 2:**
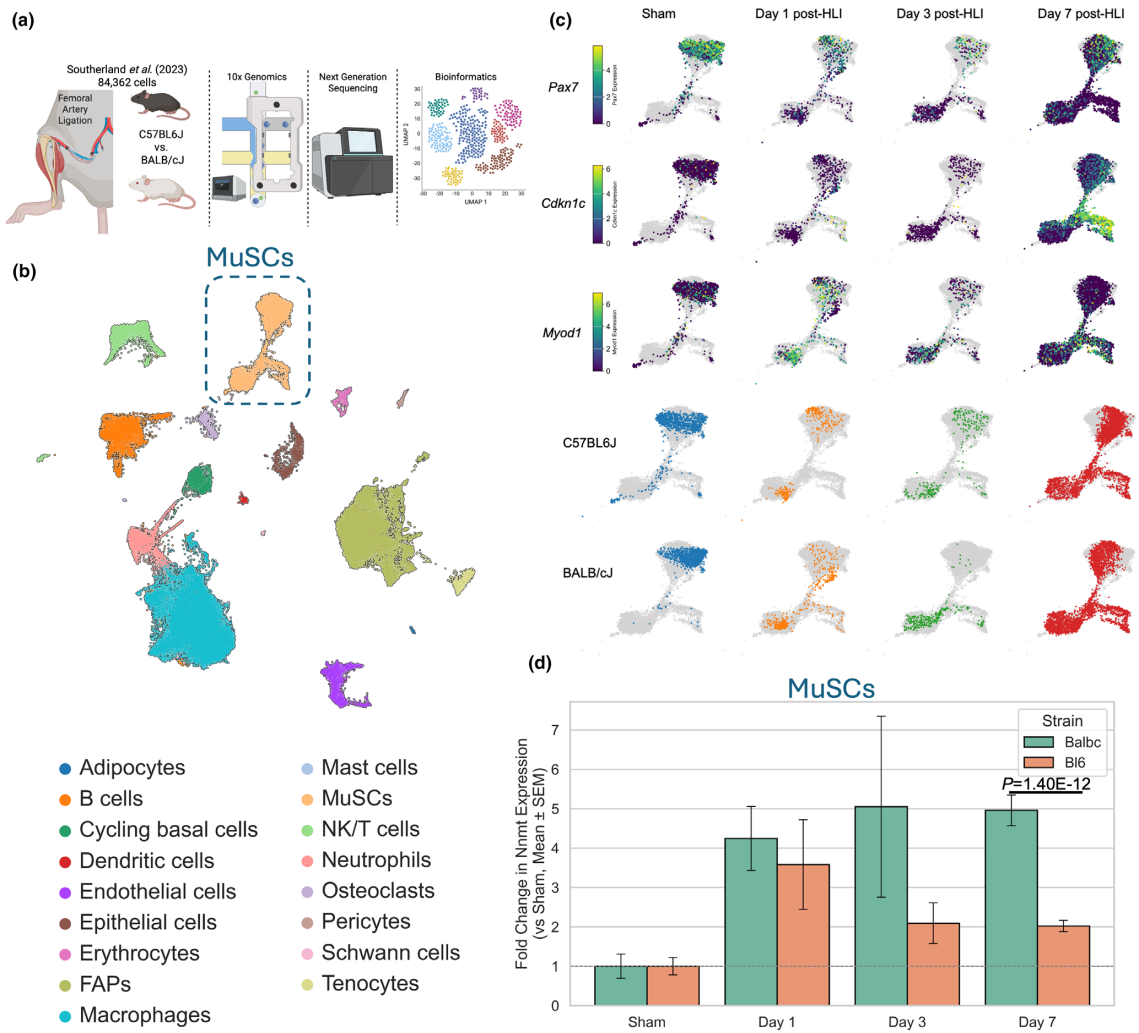
NNMT expression is persistently elevated in muscle stem cells from the ischemia‐sensitive BALB/cJ mice. (a) Graphical depiction of publicly available scRNAseq dataset and approach made with Biorender.com. (b) UMAP of the combined scRNAseq dataset containing 84,362 mononuclear cells with their annotation of cell type. (c) Presentation of MuSC cells by marker genes and strain. (d) Quantification of the relative change in *Nnmt* expression in MuSCs for both strains under sham and post‐hindlimb ischemia surgery Days 1, 3, and 7. Statistical analysis in Panel D performed using Welch's test.

Next, we took advantage of a publicly available single‐cell RNA sequencing dataset (Southerland et al., 2023) which contains cells isolated from the ischemia‐resistant C57BL6J and ischemia‐sensitive BALB/cJ mouse strains at multiple timepoints following hindlimb ischemia surgery (Figure 2a). As presented by the original authors, all major mononuclear cell types were identified in the dataset (Figure 2b), including muscle stem cells (MuSCs, otherwise known as satellite cells), endothelial cells, and fibro‐adipogenic progenitor cells (FAPs). Because a previous study demonstrated that NNMT inhibition promoted the activation of MuSCs following barium chloride injury, we explored whether strain differences in NNMT expression may be present following hindlimb ischemia. Within the MuSC cell cluster, three distinct branches were visible and feature plots of three marker genes identified quiescent (*Pax7* ^+^), activated/proliferating (*Cdkn1c* ^+^), and differentiating (*Myod1* ^+^) branches (Dumont et al., 2015; Yin et al., 2013) which were present in both strains (Figure 2c). Quantification of the relative changes in Nnmt expression from the sham condition demonstrated that both strains upregulate Nnmt expression post‐hindlimb ischemia, but that persistent hyperexpression was present only in BALB/cJ MuSCs (*p* = 1.40E‐12, Figure 2d).

### 3.3 NNMT inhibition does not alter perfusion recovery or capillary density but may reduce necrosis in male mice with experimental PAD

**FIGURE 3:**
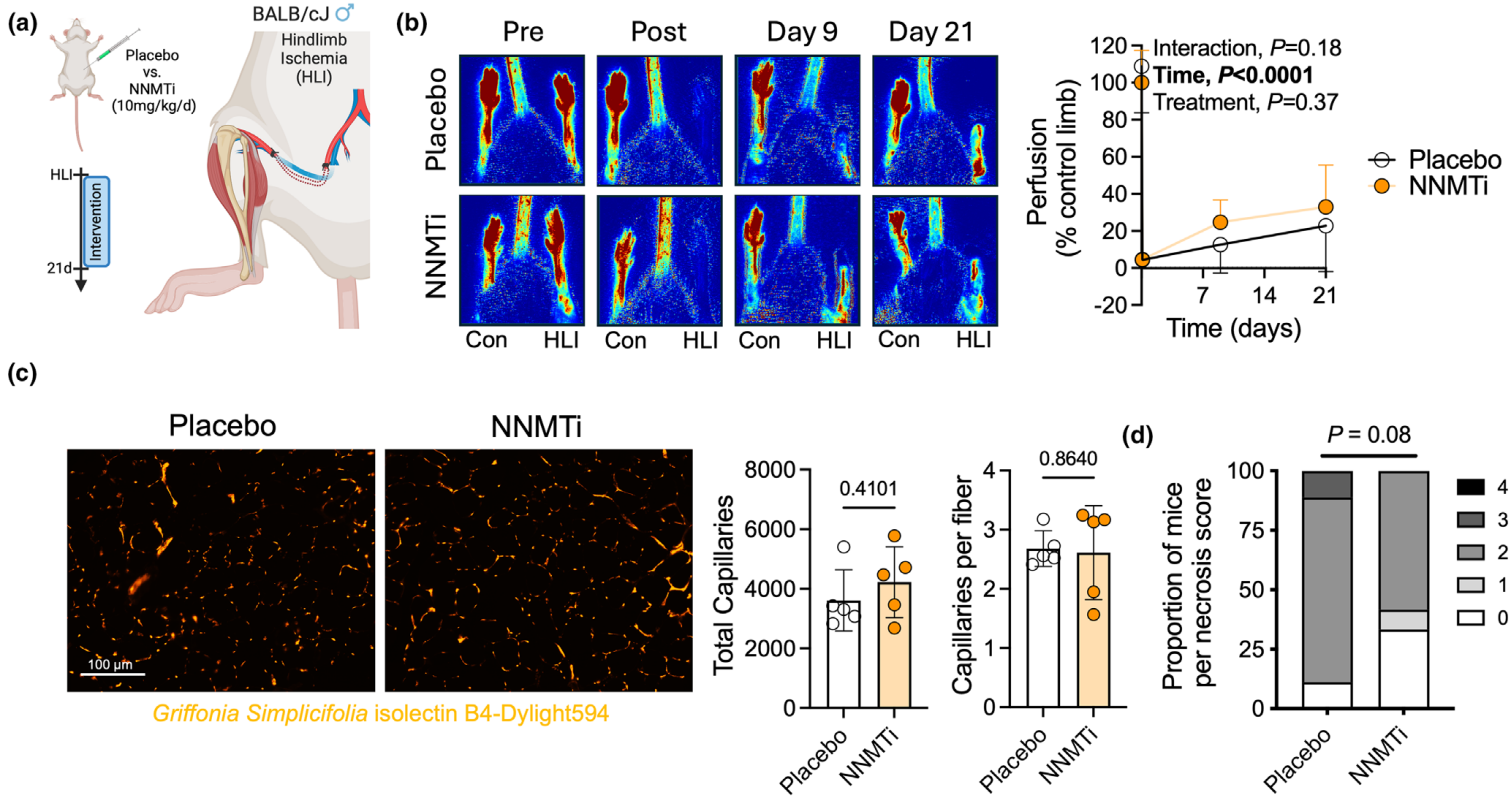
NNMT inhibition does not alter perfusion recovery or capillary density in male mice with experimental PAD. (a) Graphical depiction of the study design made with Biorender.com. (b) Representative images and quantification of laser Doppler perfusion recovery of the paw. (c) Representative images and quantification of capillary density in the ischemic gastrocnemius muscle. (d) Quantification of the proportion of mice with foot necrosis scores for severity. Panel B was analyzed using a repeated measures ANOVA. Panel C was analyzed using an unpaired, two‐tailed Student's *t*‐test. Panel D was analyzed by chi‐squared test. Error bars represent the SD.

Next, we sought to test whether inhibiting NNMT could impact the recovery from hindlimb ischemia, an experimental model of PAD, in male BALB/cJ mice. NNMTi or placebo was delivered to mice prior to surgery and once daily thereafter at a dosage of 10 mg/kg (Figure 3a). All experimenters were blinded to the treatment condition until the completion of data collection and analysis. Laser Doppler perfusion imaging demonstrated that NNMTi had no significant impact on perfusion recovery following hindlimb ischemia (Figure 3b). Consistent with laser Doppler imaging, the total number of capillaries and the number of capillaries per myofiber were not different between treatment groups (Figure 3c). Taken together, these data indicate that daily NNMTi treatment does not impact ischemic angiogenesis in muscle. The severity of foot necrosis in mice that received NNMTi treatment was not significantly different from placebo‐treated mice (*p* = 0.08, Figure 3d).

### 3.4 NNMT inhibition does not alter muscle mass or myofiber area in male mice with experimental PAD

**FIGURE 4:**
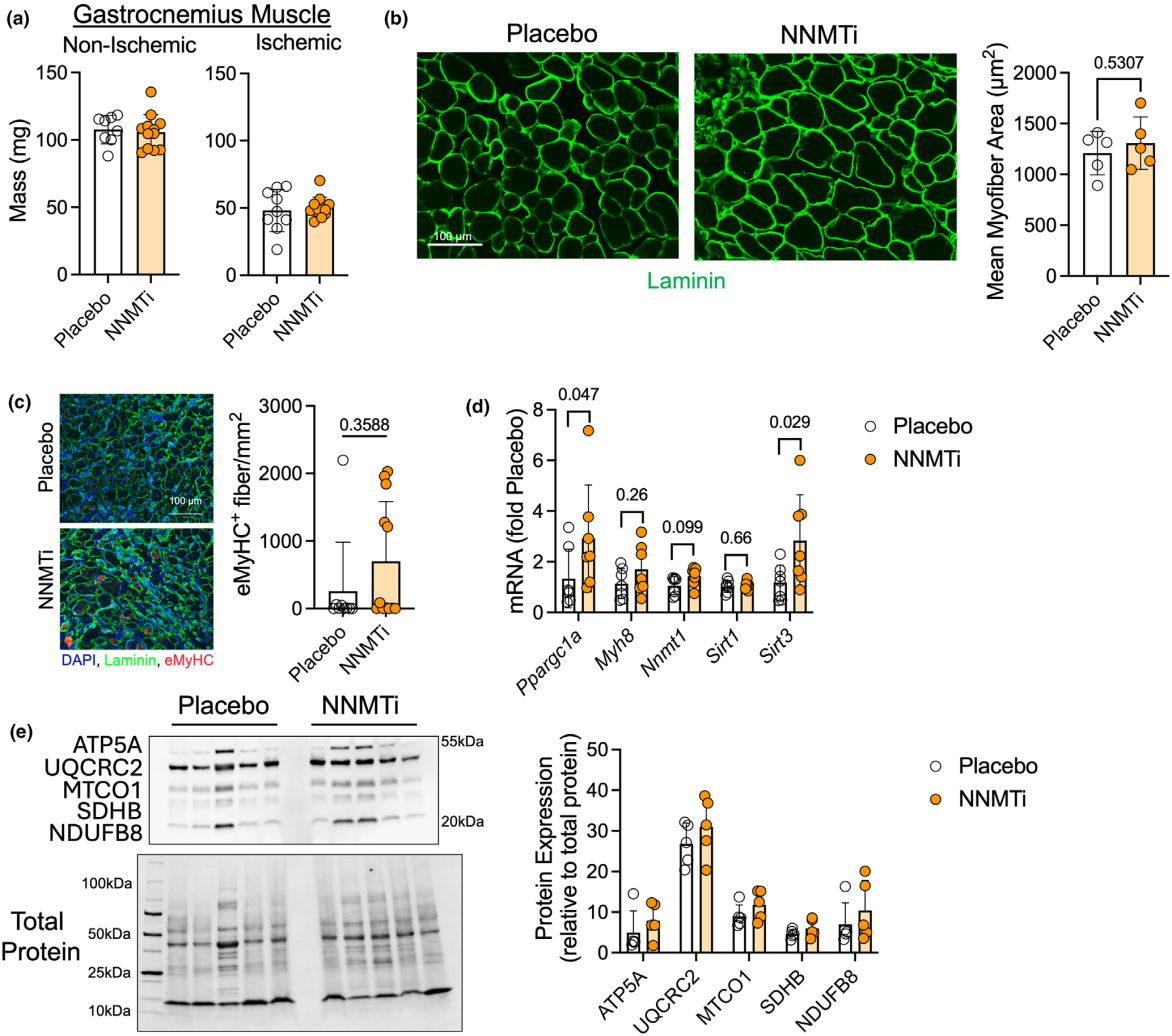
NNMT inhibition does not alter muscle mass or myofiber area in male mice with experimental PAD. (a) Mass of the non‐ischemic and ischemic gastrocnemius muscle (*N* = 9 placebo and 12 NNMTi). (b) Representative images and quantification of mean myofiber area (*N* = 5/group). (c) Representative images and quantification of embryonic myosin heavy chain (Myh3) positive fibers (*N* = 9 placebo and 12 NNMTi). (d) qPCR analysis of the ischemic muscle (*N* = 7/group). (e) Immunoblotting of mitochondrial complex proteins (*N* = 5/group). Analysis performed using unpaired, two‐tailed Student's *t*‐test. Panel C was analyzed using a Mann–Whitney test as the data were not normally distributed. Error bars represent the SD.

Regarding the impact of NNMTi on ischemic muscle, neither the mass of the non‐ischemic nor the ischemic gastrocnemius muscle was different between groups (Figure 4a). Histological analysis of the mean myofiber cross‐sectional area also showed no impact of NNMTi treatment on myofiber size (Figure 4b). Together, these data indicate that NNMT inhibition did not significantly impact muscle mass or myofiber size in male BALB/cJ mice with hindlimb ischemia. Next, we analyzed the number of myofibers expressing embryonic myosin (Myh3) and found no difference in the abundance of fibers expressing the embryonic myosin heavy chain (Figure 4c). It should be noted that muscles were harvested 21 days after hindlimb ischemia, so this analysis does not represent the early stages of muscle regeneration. mRNA analysis revealed that NNMTi‐treated mice had significantly higher expression of *Ppargc1a* and *Sirt3* when compared to placebo‐treated mice (Figure 4d). However, quantification of protein subunits found within each complex of the electron transport system indicated no differences in protein abundance in the ischemic muscle (Figure 4e).

### 3.5 NNMT inhibition significantly improves muscle strength, power, and total work in male mice with experimental PAD

**FIGURE 5:**
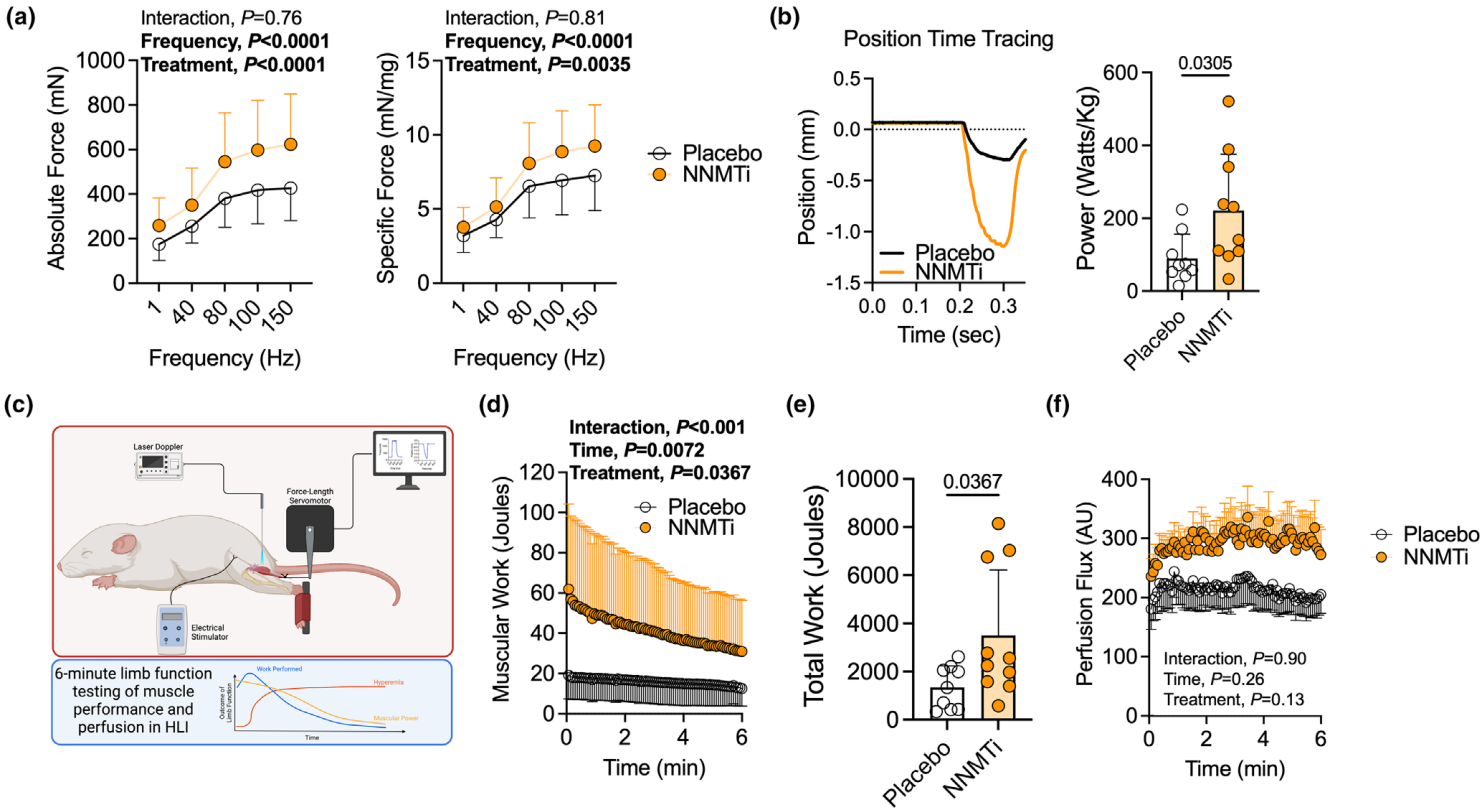
NNMT inhibition significantly improves muscle strength, power, and total work in male mice with experimental PAD. (a) Absolute and specific force‐frequency curves from the ischemic plantarflexor complex. (b) A representative position‐time tracing and quantification of muscle power output. (c) Graphical depiction of the 6‐min limb function test in mice with experimental PAD made with Biorender.com. (d) Quantification of muscular work across the 6‐min limb function test. (e) Total muscular work performed. (f) Laser Doppler flowmetry quantified hyperemia. Panel A, D, and F analyzed using two‐way ANOVA. Panels B and E analyzed using unpaired, two‐tailed Student's *t*‐test. (*n* = 9–12/group). Error bars represent the SD.

Using in situ assessments of plantarflexor muscle performance, we observed a significant treatment effect for both absolute (*p* < 0.0001) and specific force (*p* = 0.0035), demonstrating that NNMTi treatment significantly improved muscle strength and quality compared to placebo (Figure 5a). Using an isotonic shortening contraction at 30% of the maximal isometric force with 80 Hz stimulation, NNMTi treated mice exhibited significantly greater muscle power output (*p* = 0.0305, Figure 5b). Next, we performed a 6‐min limb function assessment (Figure 5c) to assess muscle performance and hemodynamics of the ischemic limb (Palzkill et al., 2025). Mice treated with NNMTi performed significantly more muscular work across the 6‐min test (Figure 5d), resulting in more total work performed (Figure 5e) when compared to placebo treated mice. Interestingly, a trending but non‐significant increase in hyperemia (*p* = 0.13) during the 6‐min limb function test was observed (Figure 5f).

## 4 DISCUSSION

Treatment options to improve lower limb function in PAD are limited. In this study, we identified elevated expression of NNMT, an enzyme that diminishes the NAD^+^ salvage pathway, in the gastrocnemius muscle of patients with PAD. Based on this, we tested whether NNMT inhibition could improve ischemic limb function in a mouse model of PAD. NNMT inhibition improved muscle strength, quality, and work output compared to placebo treatment. Remarkably, these improvements in muscular performance occurred without significant differences in perfusion or capillary density, implicating a direct effect on muscle cells that drives the therapeutic benefit of NNMT inhibition.

Our findings with NNMTi in mice with experimental PAD agree with the therapeutic benefit observed in other preclinical rodent models. For example, Neelakantan et al. used NNMTi in aged mice that received barium chloride injections to induce acute (non‐ischemic) muscle injury (Neelakantan et al., 2019). Here, the authors observed that NNMT improved MuSC function to enhance regeneration, leading to greater muscle strength. In normal aging mice, Liang et al. found that NNMT was elevated in sarcopenia conditions and that treatment with NNMTi improved grip strength in aging mice (Liang et al., 2024). The observations of Liang et al. in aging mice are supported by another study published by Dimet‐Wiley et al. (2024) who reported a ~40% increase in grip strength in aged mice treated with NNMTi compared to a placebo group. Beyond NNMT, other studies have provided evidence that modulation of the NAD^+^ salvage pathway via treatment with either NR or genetic manipulation of NAMPT can significantly enhance muscle function in a range of conditions (Fletcher et al., 2017; Frederick et al., 2016; Ryu et al., 2016; Zhang et al., 2016). Nonetheless, more studies are needed to fully understand how NAD metabolism changes in models of hindlimb ischemia and how mouse genetic strain, sex, age, and relevant comorbid conditions influence NAD homeostasis.

A recent randomized clinical trial in 90 patients with PAD reported that NR supplementation significantly improved walking performance (McDermott et al., 2024). This exciting result highlights the potential for NAD^+^ modulating therapies to improve lower limb function in PAD. Our results in a preclinical PAD model indicate that NNMTi improved limb function, but NR supplementation was not examined. Future studies should consider that NNMTi treatment could be combined with nicotinamide supplementation to augment the therapeutic effects. Beyond pharmacological treatments, exercise is another approach shown to enhance NAD homeostasis (de Guia et al., 2019) and is one of the few available treatments shown to improve walking performance in patients with PAD (McDermott, Ades, et al., 2009). Thus, a promising avenue may be the combination of NAD‐modulating treatments with exercise training to promote lower limb function in PAD. To our knowledge, only two studies have modulated the NAD pathways in hindlimb ischemia (Kiesworo et al., 2023; Kiesworo et al., 2025), both of which indicated improved angiogenic capacity. This finding contrasts with our findings with NNMTi, although we acknowledge a more thorough vascular function assessment could reveal differences not detected with laser Doppler.

This study has some limitations. First, the preclinical PAD model employed herein used only male BALB/cJ mice, which are known to be sensitive to limb ischemia and thus do not represent the entire spectrum of PAD adequately (Dokun et al., 2008; McClung et al., 2017; Ryan et al., 2020; Schmidt et al., 2017; Schmidt et al., 2018). This strain was chosen because ischemia‐resistant strains, such as C57BL6J, display rapid recovery following hindlimb ischemia (Ryan et al., 2020) such that detecting the therapeutic effect of NNMTi treatment may prove difficult. Further to this issue, the mice employed in this study were male only, young in age, and free of co‐morbid diseases such as diabetes, hypertension, hyperlipidemia, obesity, and renal disease, all of which are relatively common patient characteristics. Future studies are needed to test the efficacy of NNMTi in female mice with experimental PAD. Second, the acute nature of hindlimb ischemia does not model the progressive atherosclerotic disease development that is the most common cause of PAD in humans. However, mice don't generate atherosclerotic blockages in the blood vessels that perfused the lower hindlimb, which makes it challenging to test new therapies to improve lower limb function. Finally, we did not measure NAD^+^ levels in this study. We chose not to measure NAD metabolites because of the likely impact of performing the 6‐min limb function test prior to muscle harvest. As limb function is one of the most important clinical outcomes in patients with PAD, we felt compelled to prioritize this outcome measure in our study. Thus, separate animals would be needed to quantify the NAD metabolite pool, and the beneficial effects of NNMTi observed in this study cannot be directly linked to NAD^+^ homeostasis, and other possible off‐target effects could contribute to the improved muscle function observed. Finally, this study did not include a sham surgery group, so it was not possible to quantify the magnitude of NAD dysregulation induced by hindlimb ischemia.

In summary, this study provides evidence showing that NNMT inhibition improves ischemic limb function in male mice with experimental PAD. The improved ischemic limb function occurred without corresponding changes in limb perfusion, indicating that NNMTi does not impact ischemic angiogenesis directly or indirectly.

## CONFLICT ON INTEREST STATEMENT

None.

## FUNDING INFORMATION

This study was supported by National Institutes of Health (NIH) grants R01‐HL149704 and HL171050 (Terence E. Ryan). Gengfu Dong was supported by the American Heart Association grant 24PRE1191760.

## ETHICS STATEMENT

All procedures were approved by the Institutional Animal Care and Use Committee of the University of Florida (protocol 202400000402).
